# Comparing the performance of Kernel PCA Mix Chart with PCA Mix Chart for monitoring mixed quality characteristics

**DOI:** 10.1038/s41598-022-20122-w

**Published:** 2022-09-20

**Authors:** Muhammad Ahsan, Muhammad Mashuri, Hidayatul Khusna

**Affiliations:** Department of Statistics, Institut Teknologi Sepuluh Nopember, Surabaya, 60111 Indonesia

**Keywords:** Engineering, Mathematics and computing

## Abstract

Along with the development of information and technology, the quality characteristics of a product cannot be monitored separately in the different types of control charts. In the past, conventional control charts were developed to monitor only one type of quality characteristic. The variable control charts are used to observe the variable or metric quality characteristics. Meanwhile, in monitoring non-metric characteristics or categorical data, attribute control charts are employed. To accommodate these two types of data, the PCA Mix control chart is suggested to simultaneously monitor these two types of data in one chart. However, some drawbacks occur when this chart is applied to monitor non-metric data which has an imbalanced proportion. Therefore, the Kernel PCA Mix control chart is created to overcome the gaps that occurred in the PCA Mix chart. Similar to the previous chart, this chart is also constructed using Hotelling’s *T*^2^ statistics with Kernel Density Estimation control limit. Several simulations are used to evaluate the performance of the proposed control charts. The simulation results show that the proposed chart has a better result than the previous control chart, especially for a small mean shift with an imbalanced proportion of non-metric data. However, the PCA Mix chart has a similar performance to the proposed chart when it is applied to monitor the balanced proportion of categorical data with a large mean shift. The application with simulated data with various scenarios and the real-world case also shows that the Kernel PCA Mix chart performs better compared to the performance of the PCA Mix chart.

## Introduction

Monitoring the quality of a product is crucial in maintaining a company's reputation. In ensuring the quality provided to customers in optimal conditions control charts can be used. The control chart is used to continuously monitor the quality of the product by reducing the variability between the product using a statistical method. In its development, the control chart is divided into two categories, namely attribute and variable charts^1^. The attribute charts monitor the defect of the product in categorical data. Conversely, the numerical data can be monitored using variable-type charts.

The product quality is not only measured by the variable or attribute in different methods but also can be monitored together using a mixed attribute and variable control chart. To accommodate these needs, some researchers have studied the development of mixed characteristics charts. Aslam et al.^2^ combined the combined $\overline{X}$ and *np* charts in monitoring the quality processes. This chart is developed by transforming the variable characteristics into attributes which are then inspected together on a chart. The performance of the Aslam mixed chart is competed with Hybrid Exponential Weighted Moving Average (HEWMA)^3^ and it is found that HEWMA has effective performance for some cases. Wang et al.^4^ introduced a spatial sign covariance matrix-based chart by integrating the standardized ranks and spatial signs to estimate the mixed statistic. Furthermore, Ahsan et al. introduced the *T*^2^-based Principal Component Analysis (PCA) Mix chart to monitor the mixed characteristics processes^5^ and to detect outliers^6^ using the Kernel-based control limit^7^. However, the performance of the PCA Mix chart is decreasing while it is used to inspect the attribute data with an extremely imbalanced proportion. Whereas most processes in production have an extreme imbalanced proportion for attribute data. For instance, in the production process, 95 percent of the product has good quality while 5 percent is defective product.

To solve the issue, the Kernel PCA can be used in handling the mixed characteristics. The method was firstly developed by Schölkopf^8^. The mixed quality characteristics are combined by using the Kernel function. First, the categorical data is transformed into a dummy form, and together with the numerical data, the kernel function is formed. Further, the eigenvalue decomposition is performed in feature space and the Principal Components Scores (PCs) are calculated. Finally, the *T*^2^ statistic is estimated from the calculated PCs. Similar to the PCA Mix chart control chart, this chart also employs the Kernel Density Estimation (KDE) in calculating the control limit. Based on the problems mentioned, this paper is proposed to compare the performance of the Kernel PCA Mix chart and PCA Mix chart in detecting the mean process shift. Through the simulation process, the performance of the charts is evaluated for some scenarios. Both charts are also applied to the simulated data to determine its ability to monitor the mean process shift.

The rest of the paper is organized as follows: Sect. 2 describes the related works. The charting procedure of PCA Mix and Kernel PCA Mix chars is described in Sect. 3. Section 4 presents the performance comparison of two charts. The utilization of the proposed chart in simulated and real data is shown in Sect. 5. The conclusions and suggestions for future research are presented in Sect. 6.

### Related work

The development of multivariate variable control charts is focused on three types such as Hotelling’s *T*^2^, Multivariate EWMA, and Multivariate CUSUM charts. For Hotelling’s *T*^2^ type chart, Robust *T*^2^ control chart with median estimators ^9^ and Fast MCD ^10^ are recently developed. Haddad et al.^11^ proposed Bivariate Hotelling’s *T*^2^ charts using bootstrap data. Bivariate Hotelling’s *T*^2^ Control Chart using copula is proposed by Tiengket et al. ^12^. Ahsan et al. ^13^ proposed the PCA-based *T*^2^ control chart for monitoring the network anomalies. Moreover, the recent development of Multivariate EWMA and Multivariate CUSUM charts includes Adaptive MEWMA chart ^14^, MEWMA-CoDa chart ^15^, Max MCUSUM control chart ^16^, Dual MCUSUM charts with auxiliary information ^17^, and Residual-based Max MCUSUM for autocorrelated processes ^18^.

On the other hand, the recent development of the attribute chart is focused on the multi-attribute chart and Poisson chart. A synthetic control chart for attribute inspection is developed by Zhou, Liu, and Zheng ^19^. Mashuri et al. ^20^ proposed fuzzy bivariate for monitoring the Poisson process. The attribute chart for the joint monitoring of mean and variance is presented by Quinino et al.^21^. A multivariate Poisson chart using multiple dependent state repetitive sampling (MDSRS) has a better performance than the conventional one based on repetitive sampling^22^. Aslam, Bantan, and Khan ^23^ introduced Shewhart attribute control with the neutrosophic statistical interval. Ahsan, Mashuri, and Khusna^24^ evaluated the performance of the attribute chart for a large sample size.

Moreover, the recent development of mixed charts is still limited. Ahsan et al. developed the PCA Mix chart for monitoring the outlier^6^ and process shift ^5^. Wang et al.^4^ introduced the multivariate sign chart and found that the proposed control chart has superiority in monitoring mixed-type data. Aslam et al. proposed the mixed chart^2^ and HEWMA chart^3^ to monitor the variable and attribute characteristics.

### Ethical approval

This work does not involve experiments on animals and humans.

## Charting procedures

This section discusses the charting procedure for PCA Mix and Kernel PCA Mix chart. The procedures are given in flowchart form. The procedures of both charts are given as follows:

*PCA mix chart procedures*

Let ${\mathbf{X}}_{1}$ is a $n \times p$ matrix that consists of metric data and ${\mathbf{X}}_{2}$ is a $n \times q$ matrix that consists of non-metric data. Let ${\mathbf{G}}$ is defined as a $n \times m$ matrix of the dummy coding from each level on non-metric data, where $m$ is the number of levels in categorical variables. If ${\mathbf{Z}}_{1}$ and ${\mathbf{Z}}_{2}$ are the mean-centered matrix of ${\mathbf{X}}_{1}$ and ${\mathbf{G}}$, then the first step in calculating the principal component mixed score is creating the ${\mathbf{Z}}$ sized $n \times (p + m)$:

$${\mathbf{Z}} = [{\mathbf{Z}}_{1},{\mathbf{Z}}_{2} ].$$

The next step is forming a matrix ${\tilde{\mathbf{Z}}}$ as:

$${\tilde{\mathbf{Z}}} = N^{\frac{1}{2}} {\mathbf{ZM}}^{\frac{1}{2}},$$

where ${\mathbf{M}} = diag\left( {1,...,1,\frac{n}{{n_{1} }},...,\frac{n}{{n_{m} }}} \right)$ is the columns weights of **Z**, while the first *p* columns of **Z** are weighted by 1 and last *m* columns are weighted by $\frac{n}{{n_{s} }},$ for $s = 1,2, \ldots,m.$ and ${\mathbf{\rm N}} = \frac{1}{n}{\mathbf{I}}_{n}$ is the rows weights of **Z**. The principal components score mixed is calculated using the following equation.

$${\mathbf{Y}}^{mix} = \,{\tilde{\mathbf{Z}}\mathbf{MV}},$$

where ${\mathbf{V}}$ is $(p + m) \times r$ matrix of eigenvectors of ${\tilde{\mathbf{Z}}}$ calculated using the Generalized Singular Value Decomposition (GSVD) ^25^.

**Figure 1:**
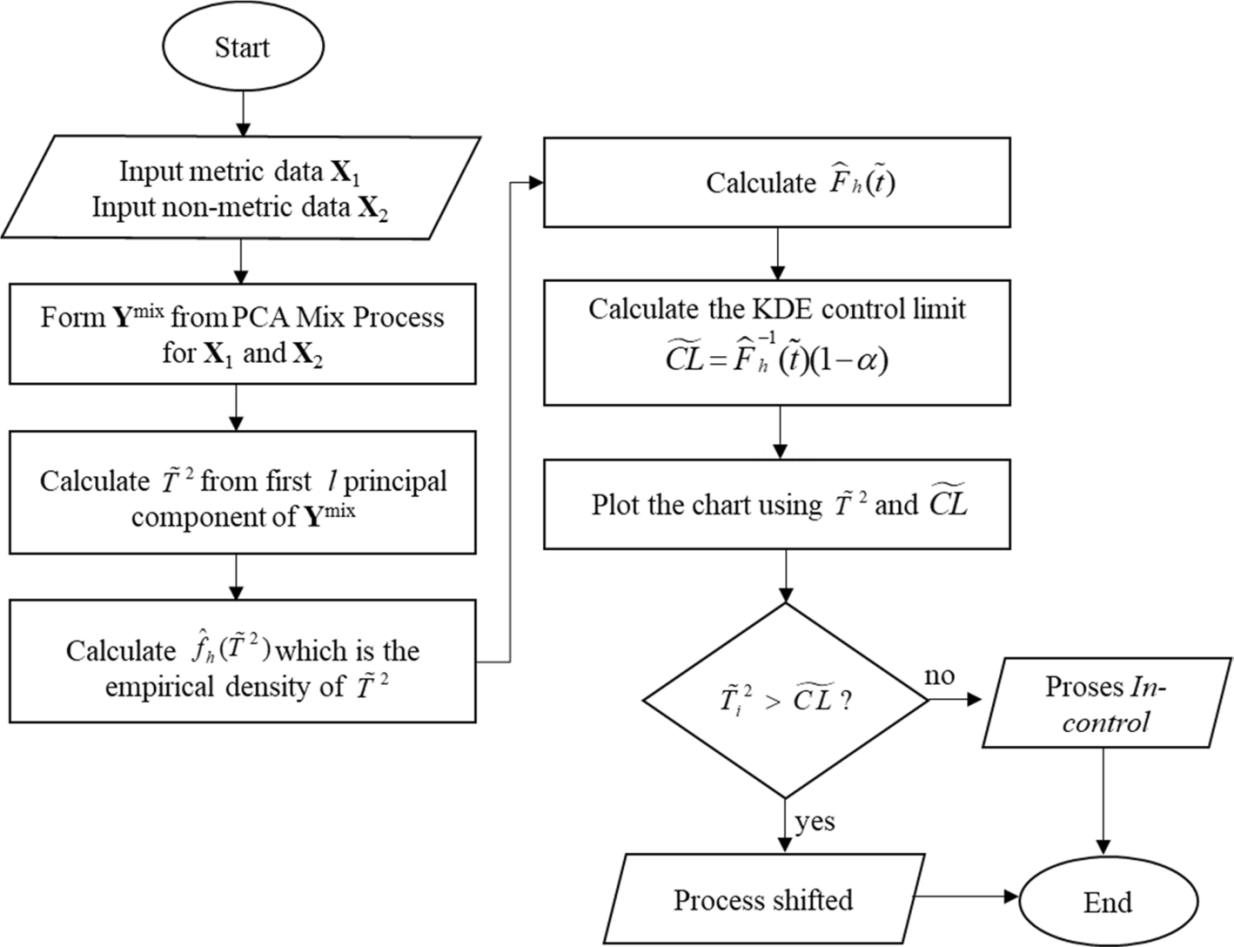
Procedures of PCA Mix chart.

Figure 1 shows the general procedures of the PCA Mix control chart. By employing the PCA Mix method ^25^, the Principal Component Scores (PCs) are formed from the mixed characteristics. Further, the $\tilde{T}^{2}.$ statistic is calculated. Finally, the control limit is estimated using the KDE method with Gaussian kernel ^26^ as follows:

$$K\left( v \right) = \frac{1}{{\sqrt {2\pi } }}\exp \left( { - \frac{1}{2}(v^{2} )} \right), - \infty < v < \infty.$$

The detailed procedure of the PCA Mix chart can be found in^5^.

### Kernel PCA mix chart procedures

To overcome the nonlinearity problem, Schölkopf et al.^8^ introduced the Kernel PCA method. The main concept of this approach is calculating the PCs in feature space by conducting a nonlinear mapping $\Phi :{\mathbb{R}}^{p} \to F,\;\;x \mapsto {\mathbf{X}}$. Let the matrix **G** be mapped to feature space *F*,$\Phi (z_{1} ),...,\Phi (z_{n} )$. The covariance matrix in feature space can be written as:

$${\mathbf{C}}^{F} = \frac{1}{n}\sum\limits_{j = 1}^{n} {\Phi ({\mathbf{z}}_{j} )\Phi ({\mathbf{z}}_{j}^{{}} )^{T} }.$$

After solving the eigenvalue problem, the eigenvector ${{\varvec{\upalpha}}}_{1},{{\varvec{\upalpha}}}_{2},....,{{\varvec{\upalpha}}}_{n}$ and eigenvalue $\lambda_{1} \ge \lambda_{2} \ge... \ge \lambda_{n}$ can be determined. The principal component score **t** is calculated by projecting $\Phi ({\mathbf{z}}_{i} )$ to eigenvector ${\mathbf{V}}_{v}$ where $v = 1,2,...,l$ as follows:

$${\mathbf{t}}_{v} = \left\langle {{\mathbf{V}}_{v},\Phi ({\mathbf{Z}})} \right\rangle = \sum\limits_{i = 1}^{n} {\alpha_{i}^{v} \left\langle {\Phi ({\mathbf{Z}}_{i} ),\Phi ({\mathbf{Z}})} \right\rangle }.$$

To solve the nonlinear mapping, the following kernel function can be applied:

$$K\left( {{\mathbf{x}},{\mathbf{y}}} \right) = \left\langle {\Phi ({\mathbf{x}}),\Phi ({\mathbf{y}})} \right\rangle$$.

**Figure 2:**
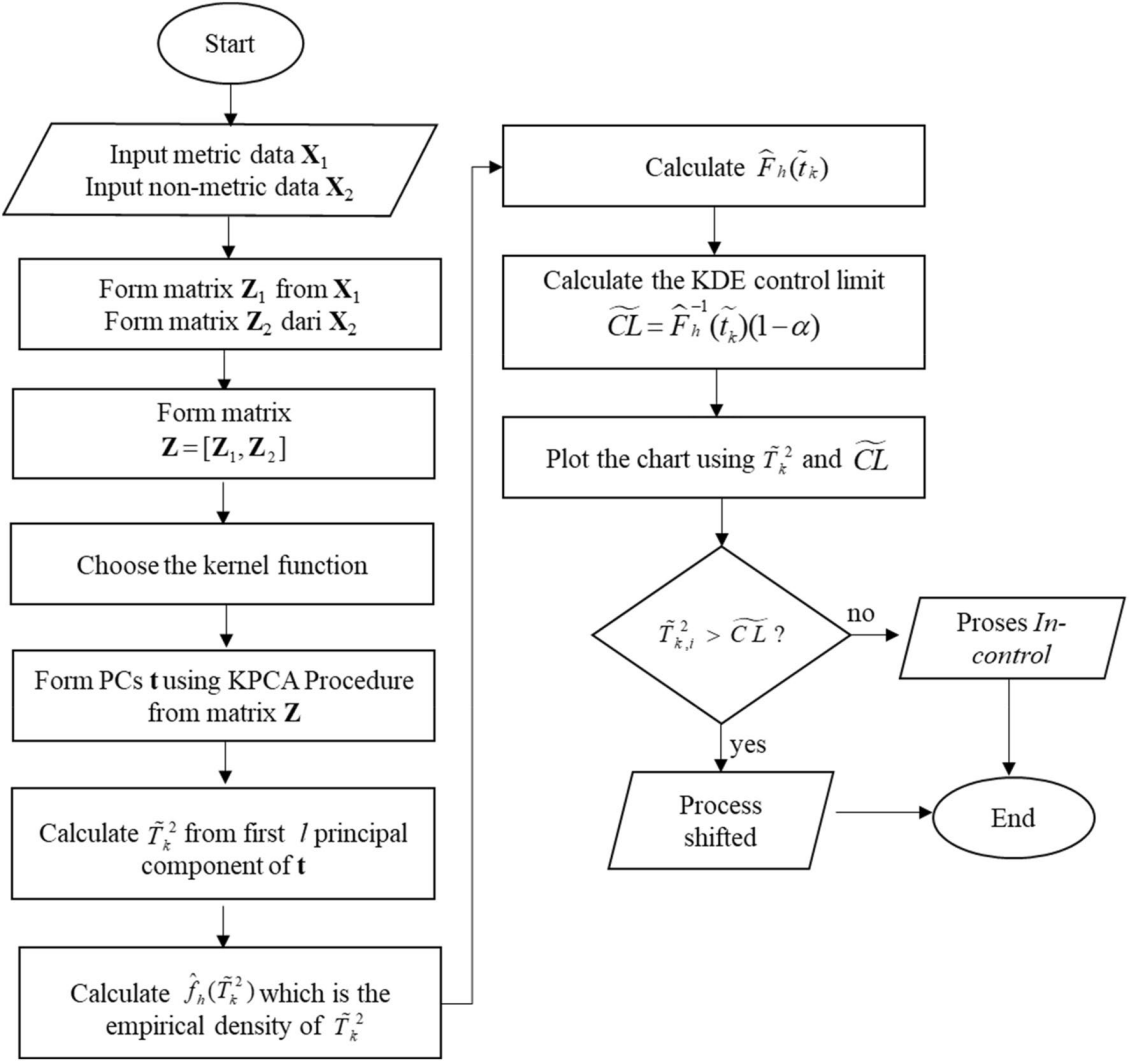
Procedures of Kernel PCA Mix chart.

Kernel PCA Mix control chart procedures are illustrated in Fig. 2. The main idea of this chart is using the Kernel PCA procedure^8^ to create the PCs by using the kernel function. In this paper, three kinds of kernel functions are used as follows:

- Linear Kernel $$K({\mathbf{x}}_{i},{\mathbf{x}}_{j} ) = \left\langle {{\mathbf{x}}_{i},{\mathbf{x}}_{j} } \right\rangle.$$
- Polynomial Kernel $$K({\mathbf{x}},{\mathbf{y}}) = \left( {\left\langle {{\mathbf{x}},{\mathbf{y}}} \right\rangle + 1} \right)^{d}$$.
- Radial Basis Function (RBF) Kernel $K({\mathbf{x}}_{i},{\mathbf{x}}_{j} ) = \exp \left( { - \sigma^{*} ||{\mathbf{x}}_{i} - {\mathbf{x}}_{j} ||^{2} } \right).$

By conducting the Kernel PCA on matrix **Z** the principal component **t** is formed. Furthermore, the statistic $\tilde{T}_{{^{k} }}^{2}$ is determined by the *l* first principal component. The final step is calculating the KDE control limit.

## Performance comparison

In this paper, simulation studies are performed to determine the performance of the PCA Mix and Kernel PCA Mix for several cases. The performance of the charts is evaluated to detect a shift in the mean process using the Average Run Length (ARL) criterion. The out-of-control ARL or ARL_1_ is estimated by adding the mean shift for each metric quality characteristic ${{\varvec{\upmu}}}_{shift} = {{\varvec{\upmu}}} + {{\varvec{\updelta}}}_{\mu }$, where ${{\varvec{\updelta}}}_{\mu } = {\mathbf{0}}{\mathbf{.1}}$.

The variable characteristics ${\mathbf{X}}_{1}$ are generated from the Multivariate Normal distribution. In this research, the number of metric quality characteristics *p* is 5. Meanwhile, the non-metric or categorical quality characteristics are generated from a multinomial distribution ${\mathbf{X}}_{2} \sim M(n,\theta_{1},\theta_{2},\theta_{3} )$ with three types of the parameter as follows:

- $$\theta_{1},\theta_{2} = 0.3{\text{ and }}\theta_{3} = 0.4$$(Balanced case)
- $$\theta_{1},\theta_{2} = 0.1{\text{ and }}\theta_{3} = 0.8$$(Imbalanced case)
- $\theta_{1},\theta_{2} = 0.05{\text{ and }}\theta_{3} = 0.9$(Extreme imbalanced case)

Tables 1, 2 and 3 present the KDE control limit and ARL_0_ for several types of non-metric data, kernel functions, and hyperparameters of kernel functions. It can be seen that for the Linear Kernel the ARL_0_ of KDE control limit is at about 370. Meanwhile, for the Polynomial kernel, *d* = 1 produces the ARL_0_ near 370. Furthermore, $\sigma^{*} = 0.001$ produces the ARL_0_ of approximately 370. Therefore, for this research, the hyperparameter used for Polynomial Kernel is 1, and the RBF kernel is 0.001.

**Table 1:** KDE Control Limit and ARL_0_ for Linear Kernel.

Case		Linear Kernel
Balanced	*p* = *5, *	10,170.3
	*l* = *2*	**375.01**
	*p* = *5, *	13,292.86
	*l* = *3*	**376.94**
	*p* = *5, *	16,007.85
	*l* = *4*	**361.19**
Imbalanced	*p* = *5, *	11,267.65
	*l* = *2*	**387.4**
	*p* = *5, *	13,567.33
	*l* = *3*	**385.82**
	*p* = *5, *	15,845.09
	*l* = *4*	**356.42**
Extreme Imbalanced	*p* = *5, *	11,217.93
	*l* = *2*	**365.19**
	*p* = *5, *	13,582.07
	*l* = *3*	**379.6**
	*p* = *5,*	15,942.24
	* l* = *4*	**376.72**

Bold values represent the ARL_0_ (In-control ARL).

**Table 2:** KDE Control Limit and ARL_0_ for several Polynomial Kernel Hyperpameters ($\sigma^{*}$).

Cases	*d*			
	1	2	3	
Balanced	*p* = *5, *	10,116.0	32,409.3	71,318.2
	*l* = *2*	**355.8**	**804.5**	**844.4**
	*p* = *5, *	13,129.4	39,586.6	83,200.8
	*l* = *3*	**358.2**	**774.8**	**815.4**
	*p* = *5, *	15,708.9	48,741.3	90,609.9
	*l* = *4*	**386.7**	**812.4**	**696.3**
Imbalanced	*p* = *5, *	10,665.1	19,383.6	57,755.2
	*l* = *2*	**371.4**	**487.7**	**755.8**
	*p* = *5, *	13,316.0	24,878.6	71,965.4
	*l* = *3*	**385.6**	**717.7**	**699.7**
	*p* = *5, *	15,864.0	29,099.0	81,079.3
	*l* = *4*	**351.6**	**656.4**	**620.3**
Extreme Imbalanced	*p* = *5, *	11,293.8	16,354.4	48,299.8
	*l* = *2*	**379.1**	**542.8**	**668.4**
	*p* = *5, *	13,476.5	22,357.9	119,229.2
	*l* = *3*	**354.4**	**787.5**	**957.1**
	*p* = *5, *	15,820.3	26,376.1	77,170.3
	*l* = *4*	**351.8**	**830.9**	**631.4**

Bold values represent the ARL_0_ (In-control ARL).

**Table 3:** KDE Control Limit and ARL_0_ for several RBF Kernel Hyperpameters ($\sigma^{*}$).

Cases		RBF Kernel Hyperparameter ($\sigma^{*}$)				
		0.001	0.005	0.01	0.05	0.1
Balanced	*p* = *5, *	10,490.0	9974.9	9358.2	6338.1	5570.2
	*l* = *2*	**388.3**	**381.7**	**418.8**	**404.7**	**512.1**
	*p* = *5, *	12,854.2	12,165.3	11,727.5	7129.1	6302.1
	*l* = *3*	**367.2**	**394.6**	**390.1**	**900.7**	**1000.0**
	*p* = *5, *	16,198.0	14,669.4	13,256.5	7799.6	6556.8
	*l* = *4*	**361.7**	**355.8**	**418.5**	**1000.0**	**904.1**
Imbalanced	*p* = *5, *	10,464.3	9642.6	9939.4	6487.4	5732.0
	*l* = *2*	**369.8**	**366.1**	**468.9**	**477.6**	**584.2**
	*p* = *5, *	12,714.6	13,059.9	11,763.3	7276.5	6496.2
	*l* = *3*	**360.0**	**385.6**	**444.6**	**963.0**	**1000.0**
	*p* = *5, *	16,821.4	14,567.6	13,399.8	7999.7	6759.7
	*l* = *4*	**386.1**	**359.7**	**406.9**	**1000.0**	**1000.0**
Extreme Imbalanced	*p* = *5, *	11,379.4	10,474.8	9693.1	6445.4	5714.9
	*l* = *2*	**386.2**	**385.9**	**358.3**	**463.5**	**687.8**
	*p* = *5, *	13,525.0	12,872.4	11,690.7	7353.6	6393.0
	*l* = *3*	**361.8**	**443.4**	**436.5**	**1000.0**	**1000.0**
	*p* = *5, *	15,612.0	14,734.5	14,882.0	7874.7	6690.3
	*l* = *4*	**354.4**	**397.2**	**437.9**	**1000.0**	**1000.0**

Bold values represent the ARL_0_ (In-control ARL).

**Figure 3:**
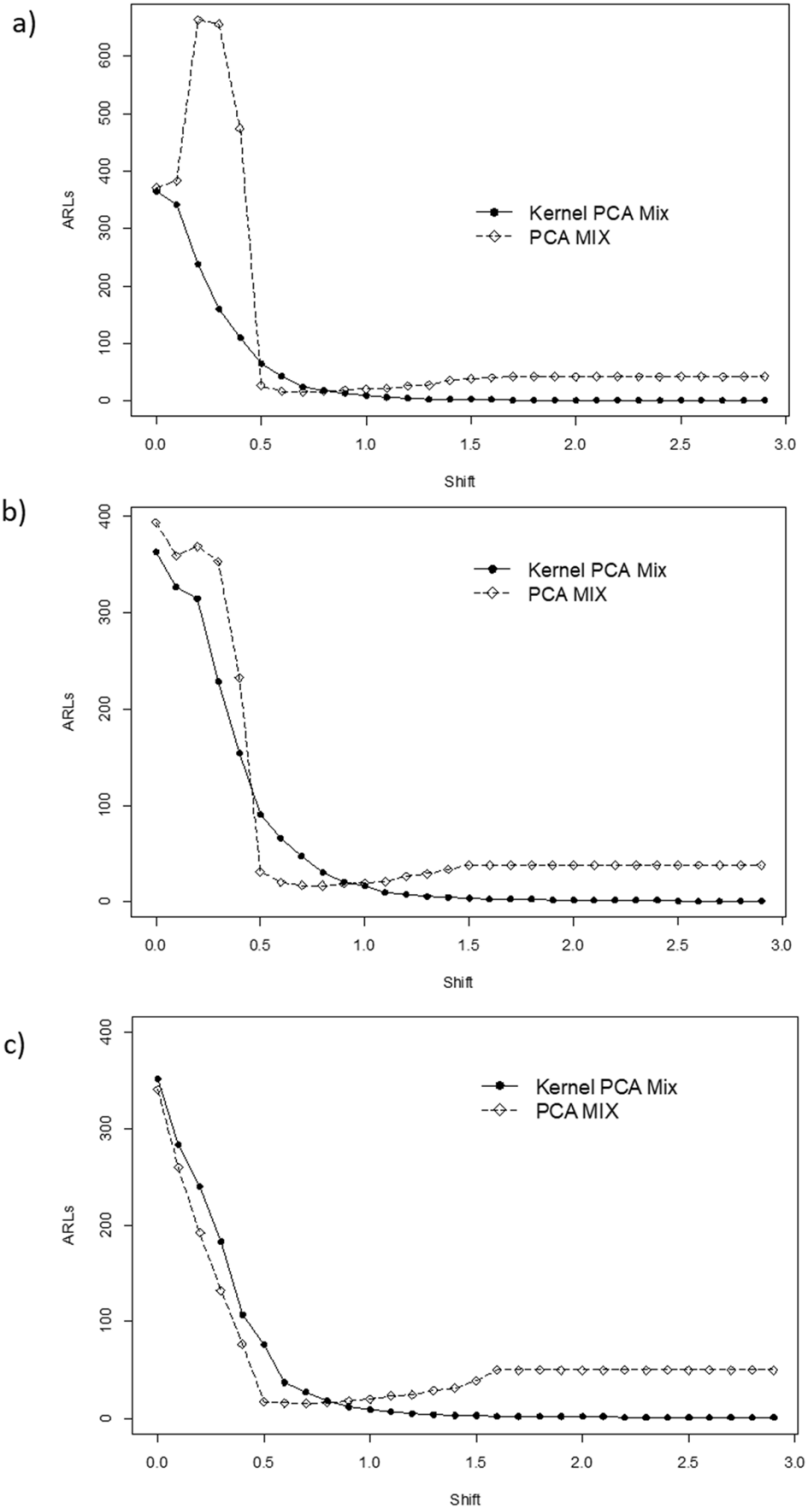
ARLs comparison between Kernel PCA Mix and PCA Mix charts with an extreme imbalanced parameter of attribute characteristic for: (**a**) *l* = 2, (**b**) *l* = 3, and (**c**) *l* = 4.

**Figure 4:**
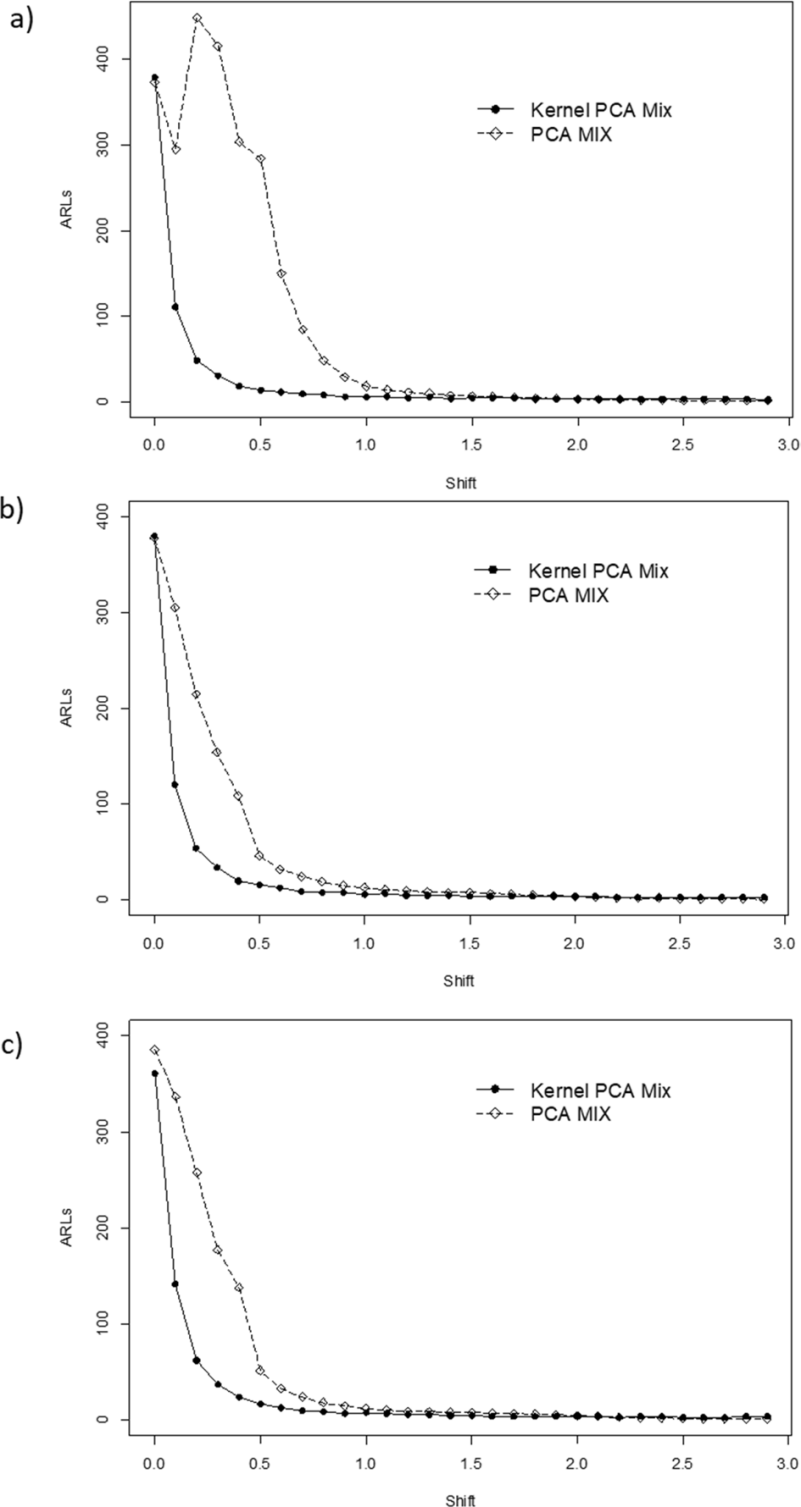
ARLs comparison between Kernel PCA Mix and PCA Mix charts with an imbalanced parameter of attribute characteristic for: (**a**) *l* = 2, (**b**) *l* = 3, and (**c**) *l* = 4.

**Figure 5:**
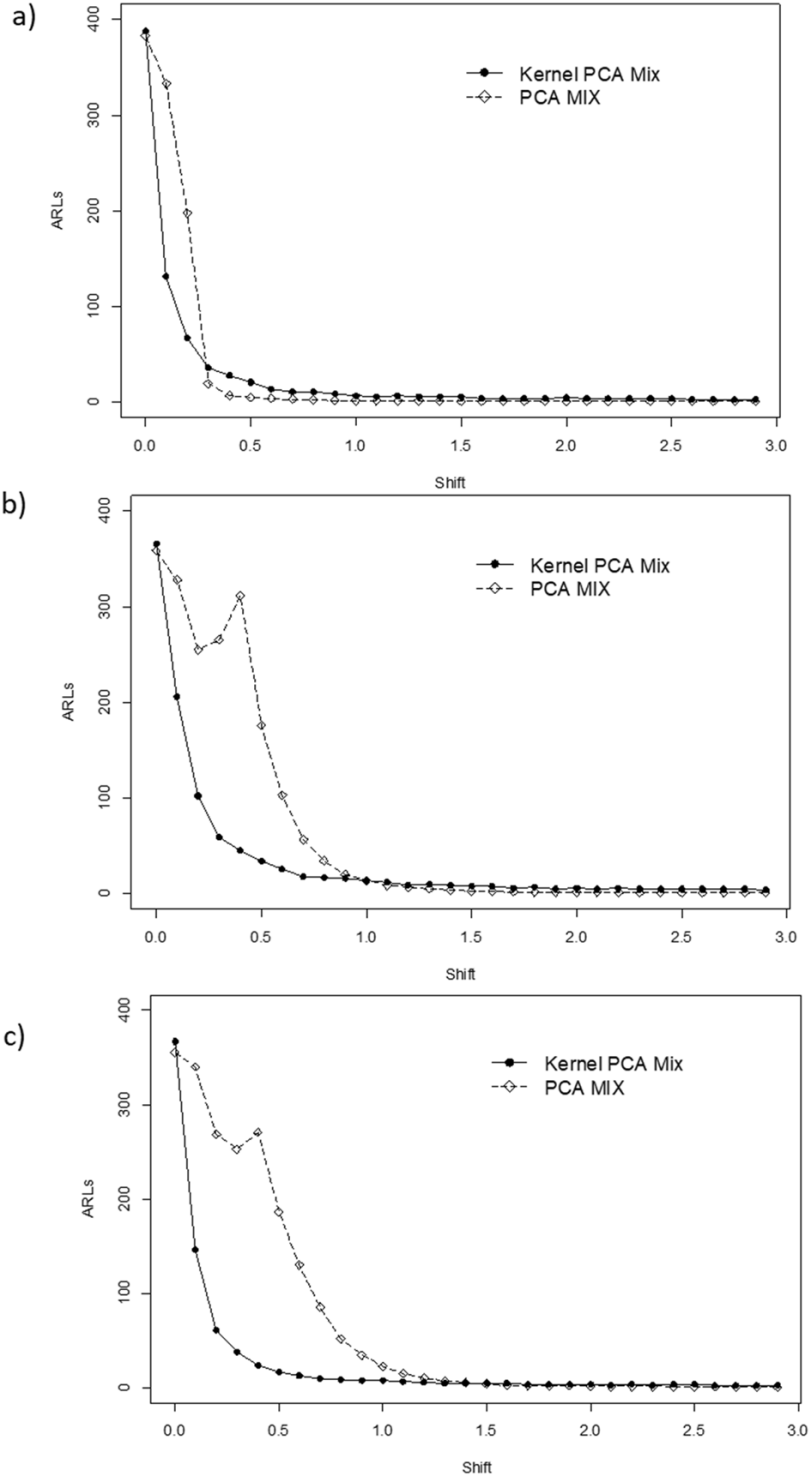
ARLs comparison between Kernel PCA Mix and PCA Mix charts with a balanced parameter of attribute characteristic for: (**a**) *l* = 2, (**b**) *l* = 3, and (**c**) *l* = 4.

Figure 3, 4 and 5 present the ARLs comparison between PCA Mix and Kernel PCA Mix chart. For extreme imbalanced. In general, from the figures, it can be seen that the Kernel PCA Mix chart yields better results compared to the PCA Mix chart. The summary of performance evaluation between the two charts is tabulated in Table 4. The sign ● represents better performance for the small mean shift while sign ⁂ represents the better performance for the large mean shift. From the table, can be seen that for small mean shifts the *T*^2^ control chart based on Kernel PCA Mix has better performance for a balanced and imbalanced parameter of the attribute characteristics. Meanwhile, for an extreme imbalanced case, the PCA Mix chart is slightly better than the Kernel PCA Mix chart in monitoring small mean shifts. Furthermore, in general Kernel PCA Mix chart with a polynomial kernel have better performance for small and large mean shifts.

**Table 4:** Summary of Kernel PCA Mix and PCA Mix performance.

Parameter of attribute characteristics	*l*	Kernel PCA Mix			PCA Mix
		RBF(0.001)	Poly(1)	Linear	
Balanced	2		⁂●		●
	3		⁂●	⁂●	●
	4	⁂●	⁂●		●
Imbalanced	2		●⁂		●
	3		●⁂	●⁂	●
	4		●⁂	●⁂	●
Extreme Imbalanced	2	●		●	⁂
	3	●	●		⁂
	4			●	⁂

## Applications

### Application to synthetic dataset

In this section, both kernel PCA Mix and Kernel PCA Mix charts are applied to the simulated data with several scenarios as presented in Table 5. The linear, polynomial, and RBF kernel are employed in this application. The first 70 data are generated to follow the multivariate normal distribution with ${{\varvec{\upmu}}} = {\mathbf{0}}$ and ${{\varvec{\Sigma}}} = {\mathbf{I}}$. On the other hand, the remaining 30 observations are generated to follow a multivariate normal distribution with ${{\varvec{\upmu}}}_{shift} = {\mathbf{2}}$ and ${{\varvec{\Sigma}}} = {\mathbf{I}}$. Furthermore, the non-metric data is generated to follow the multinomial distribution with certain parameters given in Table 2.

**Table 5:** Scenarios of simulated data for proposed chart application.

Scenario	$\theta_{1}$	$\theta_{2}$	$\theta_{3}$	*p*	*l*
1	0.30	0.30	0.40	5	4
2	0.10	0.10	0.80	5	4
3	0.05	0.05	0.90	5	4

**Figure 6:**
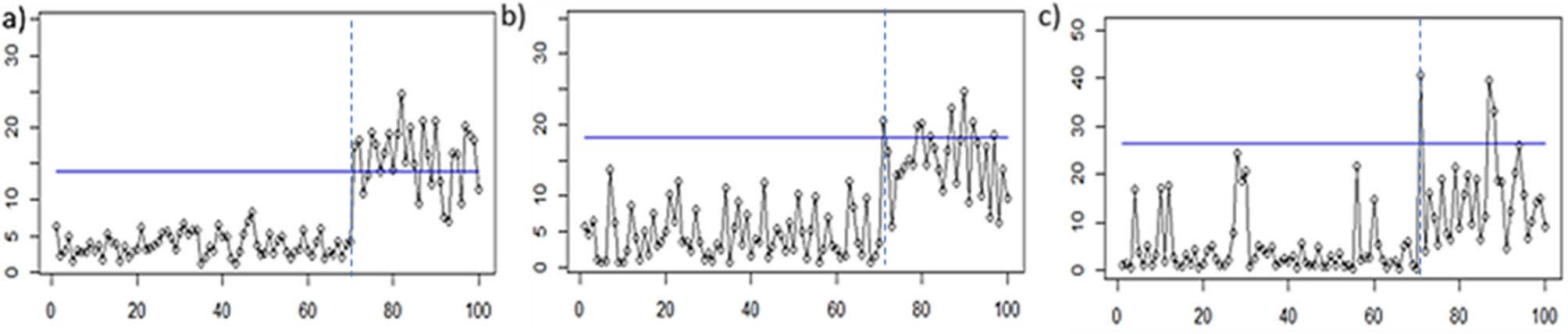
Application of the PCA Mix chart for: (**a**) the first scenario, (**b**) the second scenario, and (**c**) the third scenario.

**Figure 7:**
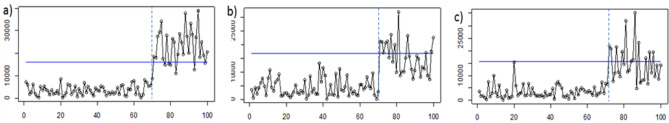
Application of the proposed Kernel PCA Mix chart with kernel RBF(0.001) for: (**a**) the first scenario, (**b**) the second scenario, and (**c**) the third scenario.

**Figure 8:**
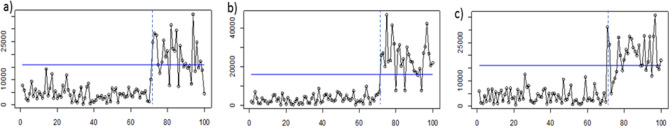
Application of the proposed Kernel PCA Mix chart with kernel Polynomial (1) for: (**a**) the first scenario, (**b**) the second scenario, and (**c**) the third scenario.

**Figure 9:**
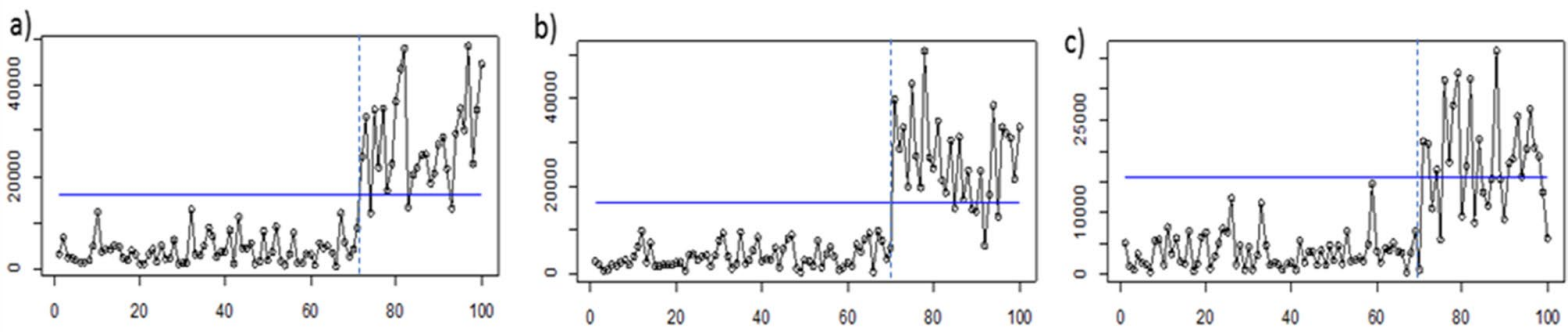
Application of the proposed Kernel PCA Mix chart with kernel Linear for: (**a**) the first scenario, (**b**) the second scenario, and (**c**) the third scenario.

The monitoring result of the PCA Mix chart is depicted in Fig. 6. From the figure, it can be seen that the PCA Mix chart has a poor performance for the extreme imbalanced attribute characteristics. Meanwhile, Figs. 7, 8 and 9 illustrate the application of the proposed chart to monitor simulated data for RBF, Polynomial, and Linear Kernels, respectively. From the results, it can be seen that for all kernel functions used, the proposed chart can correctly detect the shift in the 71st observation. Thus, it can be concluded that the Kernel PCA Mix chart has a better performance than the PCA Mix chart in monitoring the simulated data in this study.

### Application to real case data

In this subsection, the performance of two charts is compared to monitor the machine failure data (see^5^ for detailed information about the dataset). The number of observations is 250 with three of them is labeled as out-of-control observations. Table 6 presents the performance comparison between the proposed Kernel PCA Mix and PCA Mix charts in monitoring the machine failure dataset. Based on the performance evaluation, it can be seen that the Kernel PCA Mix chart using the RBF kernel can detect all out-of-control observations.

**Table 6:** Performance comparison between the Kernel PCA Mix and PCA Mix charts in monitoring the real dataset.

Criteria	Methods			
	PCA Mix Chart	Kernel PCA Mix Chart with RBF kernel	Kernel PCA Mix Chart with Polynomial kernel	Kernel PCA Mix Chart with Linear kernel
Success in detecting of in-control observations	247	246	245	245
Success in detecting out-of-control observations	2	3	1	1
Fail in detecting in-control observations	0	1	2	2
Fail in detecting out-of-control observations	1	0	3	3

## Conclusions

This research compares the capabilities of two mixed charts, the Kernel PCA Mix chart, and the PCA Mix chart, in detecting mean process shifts. Based on the ARLs results, it can be concluded that Kernel PCA Mix has better performance for a balanced and imbalanced parameter of the attribute characteristics. On the other hand, for a small mean shift with extreme imbalanced attribute characteristics, the PCA Mix chart outperforms the performance of the Kernel PCA Mix chart. When both charts are used to monitor the generated data, the Kernel PCA Mix chart surpasses the performance of the PCA Mix chart for imbalanced attribute characteristics. An application to monitor real-world cases shows that the Kernel PCA Mix chart has better performance. Further, the bootstrap resampling method can be used to estimate the control limit of the charts as demonstrated by reference^27–29^.

## Supplementary Information

Supplementary Information.

## Data Availability

The dataset is attached as a supplementary file.
